# Supplementary material for: Obesity during Pregnancy in the Horse: Effect on Term Placental Structure and Gene Expression, as Well as Colostrum and Milk Fatty Acid Concentration
Source: Vet Sci. 2023 Dec 4;10(12):691. doi: 10.3390/vetsci10120691 (PMC10748288; doi:10.3390/vetsci10120691)
Supplement: Supplementary file 1 [file vetsci-10-00691-s001.zip › vetsci-2706978-supplementary.pdf]

Table S1: Feto-placental biometry results

Results are expressed as median [Q1-Q3]. P-values were calculated using a permutation Anova (package lmperm, function aovp, R software) considering maternal age and maternal withers' height as co-variates.

Variable	N group (n=10)	O group (n=14)	p-value
Placental weight (kg)	3.47 [2.82-3.54]	3.36 [2.78-3.78]	0.706
Placental volume (L)	2.73 [2.54-3.27]	2.93 [2.37-3.12]	0.784
Placental surface (cm^2)	8933 [8529-9246]	8808 [8230-9603]	1.000
Foal birthweight (kg)	56.3 [52.3-59.4]	54.5 [52.1-57.9]	0.921
Placental efficiency	16.9 [16.6-18.4]	17.3 [15.2-18.9]	0.573

Table S2: Placental stereology

Results are expressed as median [Q1-Q3]. P-values were calculated using a permutation Anova (package lmperm, function aovp, R software) considering maternal age and maternal withers' height as co-variates. P-values were corrected for multiple testing using the fdr method.

Variable	N group (n=9)	O group (n=13)	p-value	Adjusted p-value
Volume of allantoic connective tissue (cm^3)	1597.5 [1338-2082.8]	1595.2 [1187.4-1820.3]	0.430	0.739
Volume of allantoic vessels (cm^3)	313.7 [290.8-354.3]	206.1 [161.9-337.8]	0.057	0.316
Volume of histotrophic trophoblast (cm^3)	92.5 [56-126.1]	93.4 [82.7-115.1]	0.452	0.739
Volume of haemotrophic trophoblast (cm^3)	254.2 [162.5-295.2]	337.1 [233-369.4]	0.060	0.316
Volume of microcotyledonary connective tissue (cm^3)	206.9 [180.6-242.0]	218.5 [188.9-295.0]	0.556	0.739
Volume of microcotyledonary vessels (cm^3)	245 [188.3-273.8]	318.7 [249.9-382.4]	0.248	0.578
Volume of microcotyledons (cm^3)	663.6 [562.2-788.3]	867.0 [688.3-1052.4]	0.247	0.578
Surface of allantoic connective tissue ($cm^2.10^5$)	1.3 [1.2-1.4]	1.2 [0.8-1.4]	0.099	0.347
Surface of allantoic vessels ($cm^2.10^5$)	1.2 [1-1.3]	0.9 [0.6-1.2]	0.068	0.316
Surface of histotrophic trophoblast ($cm^2.10^5$)	0.7 [0.5-0.9]	0.7 [0.5-0.8]	0.615	0.739
Surface of haemotrophic trophoblast ($cm^2.10^5$)	4.4 [3.8-4.9]	5.3 [3.8-6.6]	0.541	0.739
Surface of microcotyledonary connective tissue ($cm^2.10^5$)	3.4 [3.2-4]	3.8 [2.8-4.3]	0.804	0.804
Surface of microcotyledonary vessels ($cm^2.10^5$)	4.6 [4-6.2]	5 [4.4-6.9]	0.686	0.739
Surface of microcotyledons ($cm^2.10^5$)	11.8 [11.1-15.1]	15.6 [11-17.6]	0.667	0.739

Table S3: Placental gene expression

Results are expressed as median [Q1-Q3]. P-values were calculated using a permutation Anova (package lmperm, function aovp, R software) considering maternal age and maternal withers' height as co-variates. P-values were corrected for multiple testing using the fdr method.

Gene	N group (n=9)	O group (n=13)	p-value	Adjusted p-value
ENG	1.30 [1.02-1.60]	1.16 [1.04-1.51]	0.765	0.784
FLT1	1.39 [1.09-1.43]	1.16 [0.92-1.29]	0.745	0.784
KDR	1.19 [0.97-1.44]	1.08 [0.88-1.36]	0.510	0.784
GLUT1	1.08 [1.03-1.26]	1.21 [0.83-1.35]	0.784	0.784
GLUT3	0.87 [0.80-0.97]	0.86 [0.75-1.07]	0.092	0.784
SNAT2	1.28 [1.08-1.35]	1.09 [0.95-1.35]	0.573	0.784
CD36	1.21 [1.11-1.43]	1.33 [0.78-1.57]	0.655	0.784
LPL	0.66 [0.53-1.02]	0.97 [0.68-1.16]	0.571	0.784
H19	0.86 [0.61-1.19]	0.75 [0.56-1.05]	0.592	0.784
IGF2	1.02 [0.78-1.36]	0.71 [0.63-0.96]	0.149	0.784
IGF1R	0.91 [0.75-1.19]	0.74 [0.61-0.87]	0.448	0.784

GADPH: Glyceraldehyde 3-phosphate dehydrogenase (reference gene), SCAMP3: (Secretory Carrier Membrane Protein 3 (reference gene), RPL32: Ribosomal Protein L32 (reference gene), ENG: Endoglin (TGF-β receptor, involved in angiogenesis), Flt1: Fms Related Receptor Tyrosine Kinase 1 (VEGF receptor, involved in vasculogenesis and angiogenesis), KDR: Kinase insert Domain Receptor (VEGF receptor, involved in vasculogenesis and angiogenesis), SLC2A1: solute carrier family 2 member 1 (GLUT1, glucose transporter), SLC2A3: solute carrier family 2 member 3 (GLUT3, glucose transporter), SLC38A2: solute carrier family 38 member 2 (SNAT2, neutral amino acid transporter), CD36: Cluster of differentiation 36 (fatty acid transporter), LPL: Lipoprotein Lipase (hydrolyses triglycerides into fatty acids), H19: H19 Imprinted Maternally Expressed Transcript (IncRNA, inhibits growth), IGF-2: Insulin-like Growth Fator 2 (growth factor), IGF-1R: Insulin-like growth factor 1 receptor (IGF2 receptor, transduces IGF2 signal).

Table S4: Total fatty acid concentration

Results are expressed as median [Q1-Q3]. P-values were calculated using a permutation Anova (package lmperm, function aovp, R software) considering maternal age as covariate. P-values were corrected for multiple testing using the fdr method.

Sample	Time	N group (n=10)	O group (n=14)	p-value	Adjusted p-value
Plasma of mares (µg/mL)	Birth	1019.9 [1339.8-1541.9]	939.1 [1156-1341.8]	1.000	1.000
	30 days	497.9 [518.4-571.7]	478.2 [541.7-612.7]	0.902	1.000
	90 days	486.8 [523.8-567.8]	511.7 [584.4-645.3]	0.178	0.329
Milk (µg/mL)	Birth	3265.7 [4108.3-4491.3]	2209.3 [3065.8-5242.9]	0.764	1.000
	30 days	1200.4 [1375.4-2533.2]	1139.3 [1266.8-1548.4]	0.177	0.329
	90 days	714.3 [929.1-1059.7]	666.3 [697.1-856.8]	0.183	0.329
Plasma of foals (µg/mL)	Birth	556.4 [777.6-1036.5]	556.1 [639.3-694]	0.073	0.329
	30 days	710.9 [926.4-1004.6]	830.7 [895.9-959.2]	0.804	1.000
	90 days	723.3 [763.1-778.1]	801.8 [875.2-904.8]	0.165	1.000

Table S5: Fatty acid composition in plasma of mares during lactation (in % of total fatty acids)
Results are expressed as median [Q1-Q3]. P-values were calculated using a permutation Anova (package lmperm, function aovp, R software) considering maternal age as covariate. P-values were corrected for multiple testing using the fdr method. SFA: saturated fatty acids, MC-SFA: medium-chain saturated fatty acids, LC-SFA: long-chain saturated fatty acids, MUFA: monounsaturated fatty acids, MC-MUFA: medium-chain monounsaturated fatty acids, LC-MUFA: long-chain monounsaturated fatty acids, PUFA: polyunsaturated fatty acids.

A. At foaling

Fatty acid (% of total fatty acids)	N group (n=10)	O group (n=14)	p-value	Adjusted p-value
C10:0	0.35 [0.24-0.40]	0.33 [0.21-0.43]	0.686	0.959
C12:0	0.51 [0.49-0.71]	0.49 [0.34-0.77]	1.000	1.000
C14:0	1.67 [1.36-1.96]	1.73 [1.58-1.98]	0.686	0.959
C15:0	0.28 [0.24-0.33]	0.32 [0.30-0.33]	0.686	0.959
C16:0	20.86 [19.49-22.57]	22.41 [21.42-22.9]	0.843	0.959
C18:0	13.35 [12.02-14.23]	12.17 [11.83-12.94]	0.804	0.959
C10:1	0.51 [0.41-0.67]	0.40 [0.31-0.82]	0.784	0.959
C16:1ω7	3.13 [2.39-3.34]	3.33 [3.10-3.47]	0.342	0.959
C18:1ω7	1.23 [1.16-1.46]	1.54 [1.44-1.66]	0.275	0.959
C12:1	0.15 [0.11-0.18]	0.13 [0.11-0.15]	0.516	0.959
C14:1ω5	0.30 [0.22-0.33]	0.30 [0.26-0.37]	0.233	0.959
C15:1ω9	0.36 [0.29-0.39]	0.38 [0.28-0.52]	0.108	0.722
C16:1ω9	0.78 [0.59-0.97]	0.82 [0.74-0.90]	0.843	0.959
C18:1ω9	9.62 [8.25-10.67]	9.72 [8.89-10.72]	0.008	0.156
C20:1ω9	0.18 [0.15-0.20]	0.22 [0.2-0.22]	0.474	0.959
C18:3ω3	3.94 [3.25-4.36]	4.11 [3.75-4.91]	0.863	0.959
C20:3ω3	0.05 [0.04-0.06]	0.06 [0.03-0.08]	0.058	0.580
C20:5ω3	1.10 [0.91-1.25]	0.99 [0.66-1.22]	0.725	0.959
C18:2ω6	39.03 [36.86-43.07]	39.00 [37.28-39.94]	0.592	0.959
C20:4ω6	0.51 [0.40-0.54]	0.45 [0.37-0.50]	0.922	0.970
SFA	36.85 [35.76-38.50]	37.69 [36.59-38.2]	0.706	0.920
MUFA	16.14 [14.09-17.60]	16.54 [15.62-17.74]	0.505	0.920
PUFA	45.09 [42.93-48.09]	44.88 [43.06-45.93]	0.784	0.920
MC-SFA	2.79 [2.44-3.73]	3.08 [2.65-3.41]	0.725	0.920
LC-SFA (>C16)	33.42 [32.82-35.60]	34.66 [33.77-35.42]	0.706	0.920
MC-MUFA	1.29 [1.16-1.49]	1.20 [1.01-1.84]	0.804	0.920
LC-MUFA (>C16)	14.64 [12.52-16.61]	15.31 [13.61-16.62]	0.784	0.920
ω3 PUFA	4.96 [4.51-5.28]	5.11 [4.65-6.00]	0.961	0.961
ω6 PUFA	39.55 [37.22-43.61]	39.41 [37.83-40.39]	0.470	0.920
ω3/ω6 PUFA ratio	0.12 [0.11-0.15]	0.13 [0.11-0.16]	0.804	0.920

B. At 30 days of lactation

Fatty acid (%)	N group (n=10)	O group (n=14)	p-value	Adjusted p-value
C10:0	0.42 [0.36-0.48]	0.31 [0.27-0.49]	0.481	0.741
C12:0	0.64 [0.54-0.81]	0.52 [0.44-0.55]	0.330	0.671
C14:0	1.66 [1.43-1.99]	1.59 [1.47-1.67]	0.192	0.671
C15:0	0.36 [0.32-0.42]	0.36 [0.31-0.40]	0.941	0.941
C16:0	25.80 [25.28-28.49]	24.15 [23.4-25.26]	0.012	0.248
C18:0	14.20 [13.52-14.33]	14.11 [13.25-15.19]	0.355	0.671
C10:1	0.64 [0.53-0.81]	0.76 [0.56-0.84]	0.745	0.877
C16:1ω7	2.61 [2.55-2.82]	3.14 [2.73-3.83]	0.235	0.671
C18:1ω7	1.44 [1.27-1.73]	1.66 [1.4-1.76]	0.126	0.631
C12:1	0.21 [0.20-0.24]	0.21 [0.16-0.27]	0.804	0.893
C14:1ω5	0.29 [0.28-0.32]	0.31 [0.27-0.37]	0.706	0.877
C15:1ω9	0.53 [0.47-0.58]	0.50 [0.39-0.58]	0.241	0.671
C16:1ω9	0.44 [0.41-0.47]	0.42 [0.37-0.54]	0.394	0.671

C18:1ω9	11.57 [10.70-11.89]	12.52 [11.96-13.62]	0.03	0.304
C20:1ω9	0.25 [0.24-0.26]	0.21 [0.19-0.29]	0.126	0.631
C18:3ω3	4.57 [4.11-5.37]	4.09 [3.88-4.77]	0.603	0.808
C20:3ω3	0.09 [0.07-0.10]	0.09 [0.08-0.12]	0.606	0.808
C20:5ω3	1.00 [0.91-1.19]	1.07 [0.87-1.46]	0.307	0.671
C18:2ω6	29.39 [28.02-30.32]	30.78 [29.34-31.94]	0.403	0.671
C20:4ω6	0.34 [0.29-0.37]	0.31 [0.28-0.34]	0.922	0.941
SFA	42.84 [42.08-45.13]	41.33 [41.03-42.23]	0.038	0.225
MUFA	18.82 [18.45-19.68]	20.42 [18.75-21.29]	0.121	0.312
PUFA	36.44 [35.26-37.22]	36.87 [35.53-37.77]	0.686	0.824
MC-SFA (>C16)	3.16 [2.97-3.16]	2.84 [2.57-3.10]	0.188	0.322
LC-SFA	40.24 [39.65-42.13]	38.73 [38.36-39.33]	0.017	0.209
MC-MUFA	1.65 [1.57-1.81]	1.72 [1.46-2.02]	0.66	0.824
LC-MUFA (>C16)	16.85 [16.61-17.23]	18.56 [16.79-19.58]	0.100	0.312
ω3 PUFA	6.15 [5.51-6.84]	5.80 [5.36-6.26]	0.863	0.863
ω6 PUFA	29.7 [28.38-30.66]	31.11 [29.61-32.36]	0.400	0.600
ω3/ω6	0.20 [0.18-0.23]	0.19 [0.17-0.21]	0.863	0.863

C. At 90 days of lactation

Fatty acid (%of total fatty acids)	N group (n=10)	O group (n=14)	p-value	Adjusted p-value
C10:0	0.12 [0.07-0.15]	0.12 [0.09-0.17]	0.784	0.923
C12:0	0.32 [0.22-0.37]	0.29 [0.24-0.36]	0.581	0.923
C14:0	0.96 [0.90-1.02]	1.05 [0.85-1.22]	0.215	0.538
C15:0	0.38 [0.37-0.40]	0.38 [0.35-0.39]	0.660	0.923
C16:0	22.85 [21.44-23.97]	22.26 [21.32-23.35]	0.863	0.959
C18:0	16.64 [15.79-18.01]	16.38 [15.58-17.15]	0.564	0.923
C10:1	0.5 [0.41-0.58]	0.44 [0.35-0.53]	0.435	0.923
C16:1ω7	2.06 [1.79-2.21]	2.51 [2.06-3.16]	0.013	0.252
C18:1ω7	1.47 [1.44-1.49]	1.48 [1.39-1.63]	0.205	0.538
C12:1	0.24 [0.23-0.25]	0.24 [0.20-0.24]	0.203	0.538
C14:1ω5	0.26 [0.23-0.27]	0.28 [0.19-0.32]	0.726	0.923
C15:1ω9	0.49 [0.46-0.58]	0.49 [0.37-0.53]	0.172	0.538
C16:1ω9	0.43 [0.40-0.44]	0.47 [0.41-0.55]	0.055	0.388
C18:1ω9	12.87 [12.49-14.23]	12.83 [11.75-13.62]	0.619	0.923
C20:1ω9	0.21 [0.20-0.23]	0.18 [0.16-0.20]	0.058	0.388
C18:3ω3	3.82 [3.58-4.16]	3.87 [3.26-4.10]	0.961	0.980
C20:3ω3	0.07 [0.06-0.08]	0.07 [0.06-0.08]	0.765	0.923
C20:5ω3	0.86 [0.65-1.00]	0.87 [0.71-0.91]	0.980	0.980
C18:2ω6	33.92 [32.59-35.4]	34.6 [32.57-35.86]	0.706	0.923
C20:4ω6	0.43 [0.39-0.49]	0.46 [0.41-0.49]	0.086	0.429
SFA	41.16 [39.73-41.92]	40.82 [40.01-41.35]	0.301	0.767
MUFA	19.07 [17.89-20.08]	18.71 [17.49-20.33]	0.804	0.863
PUFA	38.92 [37.5-40.78]	39.21 [37.95-40.58]	0.765	0.863
MC-SFA	1.86 [1.52-2.08]	1.95 [1.64-1.98]	0.643	0.863
LC-SFA (>C16)	39.16 [38.23-39.93]	38.95 [38.34-39.38]	0.270	0.767
MC-MUFA	1.49 [1.34-1.72]	1.44 [1.4-1.54]	0.319	0.767
LC-MUFA ratio (>C16)	17.64 [16.72-18.67]	17.45 [16.11-19.01]	0.863	0.863
ω3 PUFA	4.73 [4.51-4.97]	4.81 [4.32-5.05]	0.843	0.863
ω6 PUFA	34.25 [33.05-35.89]	35.05 [32.97-36.34]	0.623	0.863
ω3/ω6 PUFA ratio	0.14 [0.14-0.16]	0.14 [0.12-0.16]	0.606	0.863

Figure S1: Fatty acid profile in plasma of mares during lactation
A. Biplot of principal component analysis representing the first (30.1%) and second dimensions (15.8%) for fatty acid profile at birth. B. Biplot of principal component analysis representing the first (24.2.1%) and second

dimensions (21%) for fatty acid profile at 30 days of lactation. C. Biplot of principal component analysis representing the first (30.1%) and second dimensions (15.8%) for fatty acid profile at 90 days of lactation. Variables included in the analysis are represented by the black arrows. Supplementary variables are represented by the blue arrows. Milk samples from Normal mares are represented by the grey points and ellipse while milk samples from Obese mares are represented by the red points and ellipse. Ellipses represented are 95% confidence ellipses around the barycenter. SFA: saturated fatty acids, MC-SFA: medium-chain saturated fatty acids, LC-SFA: long-chain saturated fatty acids, MUFA: monounsaturated fatty acids, MC-MUFA: medium-chain monounsaturated fatty acids, LC-MUFA: long-chain monounsaturated fatty acids, PUFA: polyunsaturated fatty acids, w3= omega-3 fatty acids, w6: omega-6 fatty acids, w3.w6: omega-3/omega-6 fatty acids ratio.

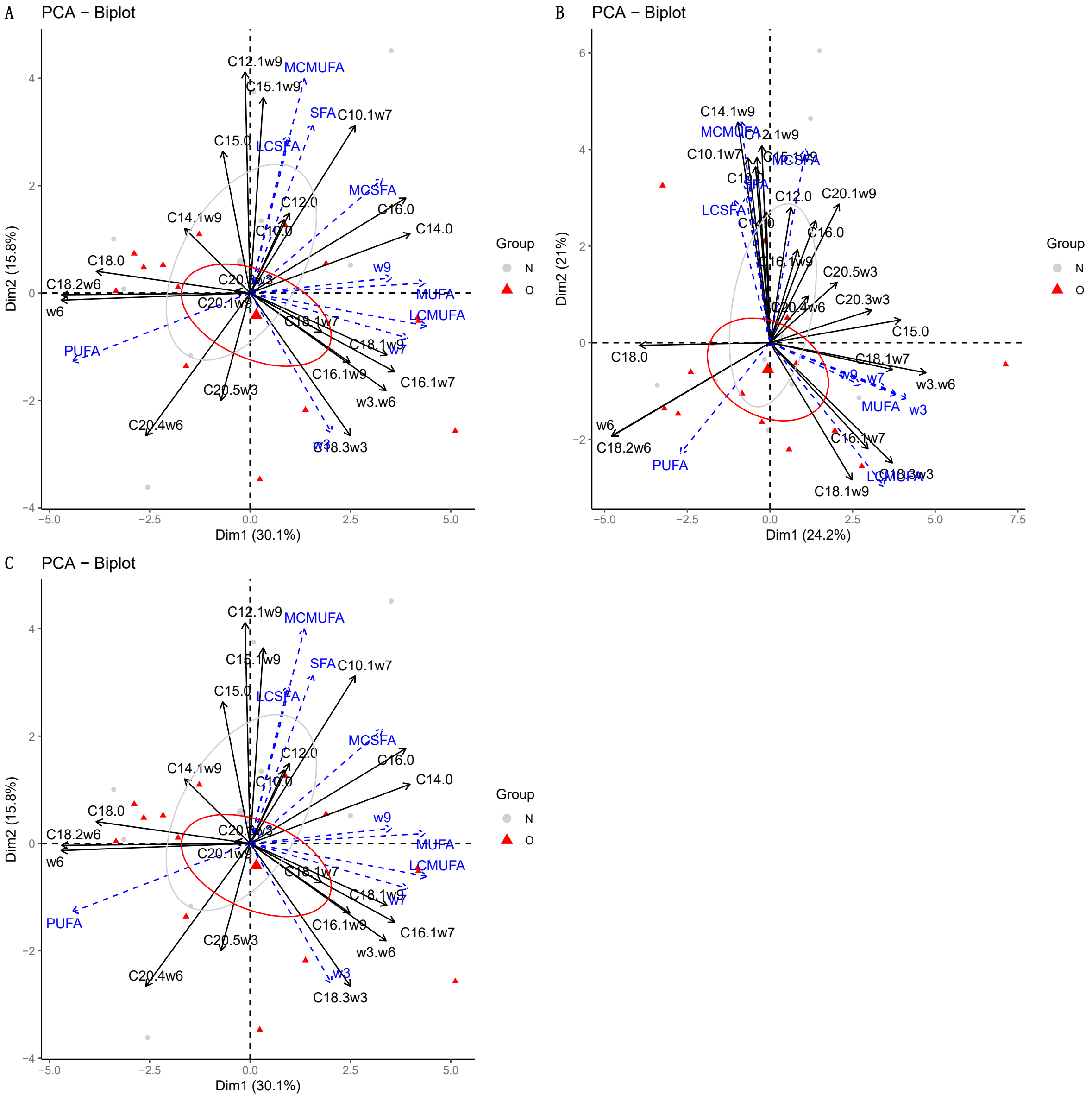

Table S6: Fatty acid profile in colostrum and milk during lactation

Results are expressed as median [Q1-Q3]. P-values were calculated using a permutation Anova (package lmperm, function aovp, R software) considering maternal age as covariate. P-values were corrected for multiple testing using the fdr method. SFA: saturated fatty acids, MC-SFA: medium-chain saturated fatty acids, LC-SFA: long-chain saturated fatty acids, MUFA: monounsaturated fatty acids, MC-MUFA: medium-chain monounsaturated fatty acids, LC-MUFA: long-chain monounsaturated fatty acids, PUFA: polyunsaturated fatty acids.

A. Colostrum

Fatty acid (%)	N group (n=10)	O group (n=14)	p-value	Adjusted p-value
C10:0	3.49 [1.05-5.84]	0.19 [0.05-0.48]	0.000	0.000
C12:0	6.29 [2.49-9.27]	1.75 [1.00-3.45]	0.003	0.013
C14:0	7.06 [6.13-8.47]	6.46 [5.41-7.79]	0.181	0.292
C15:0	0.51 [0.50-0.57]	0.53 [0.49-0.58]	0.462	0.606
C16:0	29.67 [27.85-33.66]	32.45 [31.79-33.52]	0.029	0.066
C18:0	2.09 [1.99-2.24]	2.03 [1.89-2.29]	0.961	0.961
C10:1	0.28 [0.15-0.57]	0.07 [0.03-0.10]	0.000	0.000
C16:1ω7	6.85 [6.58-7.35]	8.49 [7.77-8.99]	0.008	0.025
C18:1ω7	1.80 [1.66-1.90]	2.25 [2.00-2.38]	0.001	0.006
C12:1	0.09 [0.06-0.15]	0.06 [0.04-0.07]	0.004	0.014
C14:1ω5	0.33 [0.30-0.40]	0.33 [0.28-0.37]	0.563	0.695
C15:1ω9	0.06 [0.04-0.09]	0.04 [0.03-0.07]	0.256	0.365
C16:1ω9	1.28 [1.20-1.41]	1.47 [1.34-1.58]	0.015	0.039
C18:1ω9	20.64 [17.43-22.48]	22.96 [21.87-23.71]	0.032	0.066
C20:1ω9	0.41 [0.38-0.50]	0.43 [0.35-0.49]	0.843	0.885
C18:3ω3	8.35 [7.65-8.98]	9.00 [8.64-9.99]	0.261	0.365
C20:3ω3	0.19 [0.17-0.22]	0.18 [0.14-0.22]	0.667	0.778
C20:5ω3	0.10 [0.08-0.16]	0.11 [0.06-0.18]	0.765	0.845
C22:5ω3	0.02 [0.02-0.02]	0.03 [0.02-0.03]	0.062	0.108
C18:2ω6	7.87 [7.72-8.09]	9.11 [8.68-9.74]	0.003	0.013
C20:4ω6	0.03 [0.02-0.03]	0.02 [0.01-0.03]	0.052	0.099
SFA	50.56 [46.60-54.35]	45.03 [43.50-46.16]	0.001	0.007
MUFA	31.26 [28.95-33.91]	35.68 [34.83-37.31]	0.009	0.018
PUFA	17.09 [15.51-18.58]	19.11 [16.89-19.70]	0.052	0.063
MC-SFA	17.92 [10.25-24.74]	9.37 [6.99-12.10]	0.002	0.007
LC-SFA (>C16)	31.80 [29.69-35.76]	34.57 [33.62-35.80]	0.032	0.043
MC-MUFA	0.76 [0.57-1.23]	0.51 [0.46-0.61]	0.008	0.018
LC-MUFA (>C16)	30.89 [27.51-33.86]	35.40 [34.02-37.03]	0.012	0.021
ω3 PUFA	8.75 [7.90-9.33]	9.34 [8.86-10.32]	0.257	0.257
ω6 PUFA	7.89 [7.37-8.11]	9.14 [8.70-9.74]	0.002	0.007
ω3/ω6 PUFA ratio	1.09 [1.03-1.32]	1.07 [0.96-1.23]	0.125	0.137

B. At 30 days of lactation

Fatty acid (%)	N group (n=10)	O group (n=14)	p-value	Adjusted p-value
C10:0	1.60 [0.63-2.82]	2.64 [1.79-3.77]	0.342	0.884
C12:0	5.99 [4.50-10.14]	8.15 [6.40-11.45]	0.473	0.884
C14:0	8.28 [7.32-11.73]	9.51 [8.50-11.62]	0.725	0.967
C15:0	0.52 [0.44-0.54]	0.50 [0.47-0.52]	0.803	0.992
C16:0	27.01 [26.76-27.54]	26.81 [26.16-27.40]	0.331	0.884
C18:0	1.54 [1.20-1.61]	1.27 [1.21-1.39]	0.382	0.884
C10:1	0.46 [0.20-0.65]	0.37 [0.32-0.73]	0.541	0.884
C16:1ω7	9.34 [8.85-10.21]	9.66 [8.81-10.93]	0.843	0.992
C18:1ω7	1.85 [1.48-2.08]	1.85 [1.61-2.16]	0.441	0.884
C12:1	0.19 [0.16-0.29]	0.25 [0.20-0.33]	0.477	0.884
C14:1ω5	0.62 [0.54-0.93]	0.72 [0.62-0.92]	1.000	1.000
C15:1ω9	0.06 [0.05-0.08]	0.06 [0.05-0.09]	0.902	1.000
C16:1ω9	0.86 [0.58-0.88]	0.68 [0.58-0.85]	0.581	0.884
C18:1ω9	20.27 [15.15-21.26]	18.00 [15.45-19.93]	0.527	0.884
C20:1ω9	0.22 [0.19-0.27]	0.18 [0.15-0.21]	0.048	0.884
C18:3ω3	10.91 [10.60-11.63]	10.95 [10.19-11.44]	0.619	0.884
C20:3ω3	0.25 [0.23-0.30]	0.21 [0.19-0.25]	0.098	0.884
C20:5ω3	0.34 [0.21-0.47]	0.32 [0.24-0.40]	0.583	0.884
C18:2ω6	7.77 [5.95-7.99]	6.70 [5.57-7.27]	0.349	0.884

C20:4ω6	0.04 [0.03-0.05]	0.04 [0.03-0.05]	1.000	1.000
SFA	45.21 [41.71-53.74]	49.41 [46.32-54.30]	0.533	0.623
MUFA	34.22 [28.94-35.58]	32.28 [27.15-34.37]	0.623	0.623
PUFA	19.33 [16.83-20.63]	18.09 [17.30-18.75]	0.510	0.623
MC-SFA	16.51 [12.82-26.01]	20.78 [17.43-26.43]	0.465	0.623
LC-SFA (>C16)	28.48 [28.11-29.14]	28.36 [27.37-28.76]	0.326	0.623
MC-MUFA	1.32 [1.04-2.12]	1.69 [1.25-1.74]	0.515	0.623
LC-MUFA (>C16)	33.35 [26.81-34.65]	31.04 [25.63-33.23]	0.623	0.623
ω3 PUFA	11.50 [10.96-12.66]	11.53 [10.77-12.02]	0.555	0.623
ω6 PUFA	7.82 [5.97-8.04]	6.74 [5.90-7.31]	0.411	0.623
ω3/ω6 PUFA ratio	1.64 [1.54-1.83]	1.77 [1.50-1.98]	0.462	0.623

C. At 90 days of lactation

Fatty acid (%)	N group (n=10)	O group (n=14)	p-value	Adjusted p-value
**C10:0**	**2.85 [1.81-3.21]**	**0.25 [0.10-0.39]**	**0.000**	**0.000**
C12:0	2.39 [1.22-3.38]	3.74 [2.66-5.41]	0.030	0.150
C14:0	8.67 [7.32-9.31]	8.71 [8.27-9.47]	0.201	0.502
C15:0	0.58 [0.54-0.62]	0.56 [0.51-0.62]	1.000	1.000
C16:0	30.01 [27.31-30.66]	27.47 [26.98-28.15]	0.040	0.150
C18:0	1.42 [1.23-1.47]	1.40 [1.18-2.06]	0.345	0.691
C10:1	0.19 [0.10-0.26]	0.37 [0.24-0.49]	0.015	0.100
C16:1ω7	11.7 [10.81-13.03]	11.9 [10.30-13.93]	0.980	1.000
C18:1ω7	2.18 [1.90-2.72]	2.14 [1.97-2.36]	0.784	0.923
C12:1	0.19 [0.15-0.27]	0.30 [0.27-0.36]	0.045	0.150
C14:1ω5	0.86 [0.78-1.04]	0.97 [0.82-1.16]	0.654	0.923
C15:1ω9	0.06 [0.05-0.07]	0.07 [0.05-0.08]	0.941	1.000
C16:1ω9	0.68 [0.50-0.90]	0.84 [0.68-0.88]	0.784	0.923
C18:1ω9	21.86 [20.19-23.21]	19.76 [18.79-21.68]	0.079	0.226
C20:1ω9	0.26 [0.22-0.38]	0.26 [0.21-0.30]	0.011	0.100
C18:3ω3	12.1 [9.74-13.03]	10.33 [9.10-11.38]	0.764	0.923
C20:3ω3	0.20 [0.17-0.27]	0.17 [0.12-0.20]	0.270	0.601
C20:5ω3	0.32 [0.28-0.42]	0.44 [0.30-0.59]	0.469	0.803
C18:2ω6	6.00 [5.34-6.97]	6.74 [5.65-7.41]	0.500	0.803
C20:4ω6	0.04 [0.03-0.05]	0.04 [0.03-0.09]	0.521	0.803
SFA	47.63 [45.44-49.93]	43.22 [41.80-45.50]	0.008	0.103
MUFA	37.64 [36.23-39.10]	37.30 [35.98-38.13]	0.385	0.660
PUFA	18.34 [16.18-20.60]	17.46 [16.58-19.67]	0.803	0.804
MC-SFA	18.08 [15.01-19.51]	14.29 [12.34-16.56]	0.030	0.184
LC-SFA (>C16)	31.26 [28.57-32.27]	28.95 [28.35-30.11]	0.294	0.624
MC-MUFA	1.32 [1.09-1.61]	1.89 [1.55-1.97]	0.079	0.304
LC-MUFA (>C16)	37.14 [35.24-38.00]	36.20 [34.25-36.64]	0.312	0.624
ω3 PUFA	13.00 [10.24-13.72]	10.87 [9.71-12.86]	0.803	0.804
ω6 PUFA	6.05 [5.38-7.00]	6.79 [5.70-7.47]	0.495	0.743
ω3/ω6 PUFA ratio	1.87 [1.62-2.52]	1.67 [1.39-2.13]	0.654	0.804

Figure S2: Fatty acid profile in milk at 30 days of lactation

Biplot of principal component analysis representing the first (46.4%) and second dimensions (12.1%). Variables included in the analysis are represented by the black arrows. Supplementary variables are represented by the blue arrows. Milk samples from Normal mares are represented by the grey points and ellipse while milk samples from Obese mares are represented by the red points and ellipse. Ellipses represented are 95% confidence ellipses around the barycenter. SFA: saturated fatty acids, MC-SFA: medium-chain saturated fatty acids, LC-SFA: long-chain saturated fatty acids, MUFA: monounsaturated fatty acids, MC-MUFA: medium-chain monounsaturated fatty acids, LC-MUFA: long-chain monounsaturated fatty acids, PUFA: polyunsaturated fatty acids, w3: omega-3 fatty acids, w6: omega-6 fatty acids, w3.w6: omega-3/omega-6 fatty acids ratio.

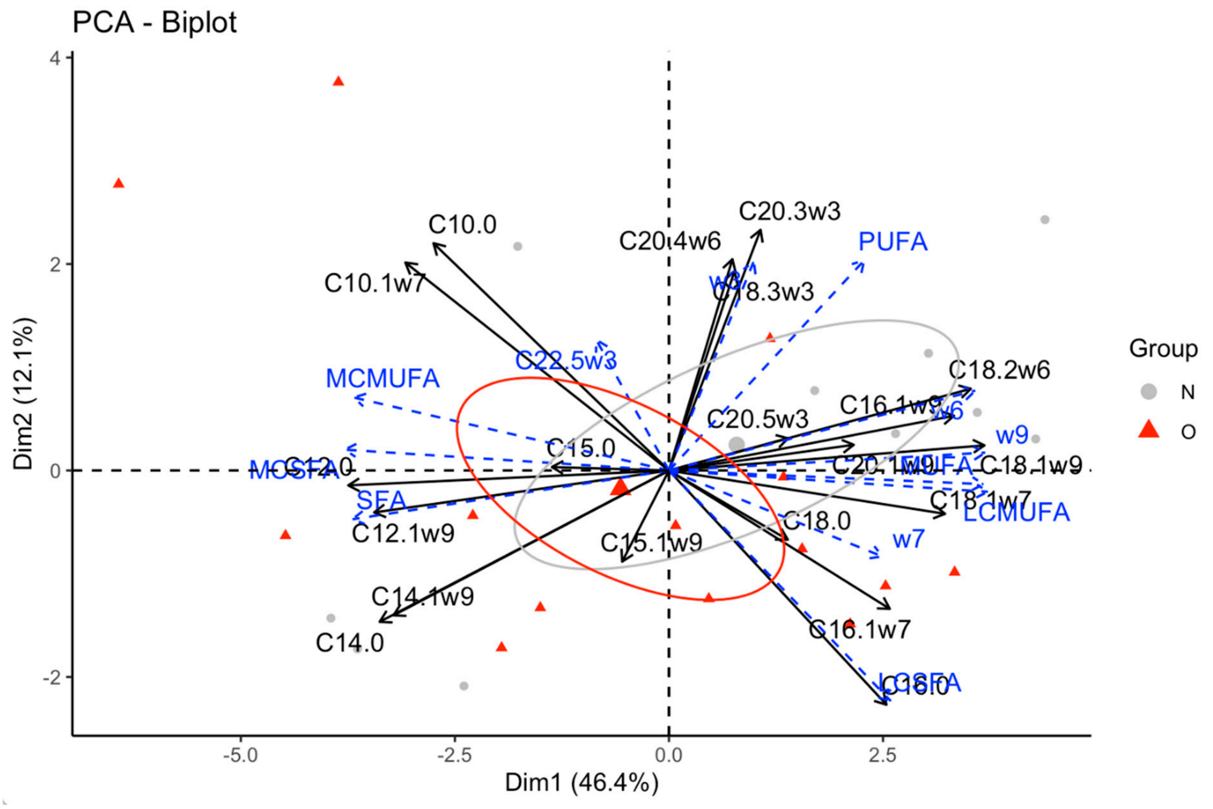

Table S7: Fatty acid profile in plasma of foals during growth

Results are expressed as median [Q1-Q3]. P-values were calculated using a permutation Anova (package lmperm, function aovp, R software) considering sex of the foal as covariate. P-values were corrected for multiple testing using the fdr method. SFA: saturated fatty acids, MC-SFA: medium-chain saturated fatty acids, LC-SFA: long-chain saturated fatty acids, MUFA: monounsaturated fatty acids, MC-MUFA: medium-chain monounsaturated fatty acids, LC-MUFA: long-chain monounsaturated fatty acids, PUFA: polyunsaturated fatty acids.

A. At birth

Fatty acid (%)	N group (n=10)	O group (n=14)	p-value	Adjusted p-value
C10:0	0.13 [0.10-0.21]	0.28 [0.24-0.39]	0.052	0.148
C12:0	0.52 [0.45-0.58]	0.52 [0.36-0.75]	1.000	1.000
C14:0	1.84 [1.68-2.08]	1.47 [1.42-1.56]	0.027	0.136
C15:0	0.23 [0.21-0.27]	0.26 [0.24-0.30]	0.123	0.246

C16:0	30.37 [29.02-30.71]	30.59 [29.37-31.97]	0.179	0.275
C18:0	9.83 [9.62-11.67]	11.53 [10.33-13.28]	0.148	0.269
C10:1	0.65 [0.59-0.73]	0.63 [0.43-0.86]	1.000	1.000
C16:1ω7	7.75 [6.12-8.47]	6.80 [5.50-7.07]	0.068	0.171
C18:1ω7	2.42 [2.11-2.58]	2.64 [2.30-3.14]	0.046	0.148
C12:1	0.20 [0.18-0.23]	0.19 [0.17-0.20]	0.784	0.980
C14:1ω5	0.18 [0.11-0.20]	0.15 [0.12-0.18]	1.000	1.000
C15:1ω9	0.21 [0.18-0.27]	0.21 [0.18-0.25]	0.941	1.000
C16:1ω9	0.98 [0.84-1.15]	0.84 [0.80-0.92]	0.095	0.212
C18:1ω9	24.68 [23.45-27.09]	24.21 [22.53-26.55]	0.353	0.504
C20:1ω9	0.18 [0.15-0.20]	0.22 [0.21-0.24]	0.012	0.113
C18:3ω3	0.48 [0.43-0.59]	0.28 [0.25-0.39]	0.017	0.113
C20:3ω3	0.65 [0.61-0.72]	0.80 [0.76-0.84]	0.013	0.113
C20:5ω3	1.26 [1.18-1.36]	1.04 [0.85-1.13]	0.045	0.148
C18:2ω6	11.97 [11.04-13.44]	13.20 [11.79-13.5]	0.169	0.275
C20:4ω6	1.54 [1.30-1.79]	1.55 [1.12-1.71]	0.660	0.881
SFA	43.43 [41.66-44.84]	45.19 [45.00-47.02]	0.008	0.032
MUFA	37.52 [33.84-40.33]	35.53 [34.34-37.78]	0.363	0.623
PUFA	27.08 [26.11-27.60]	27.60 [26.79-27.89]	0.433	0.649
MC-SFA	2.89 [2.60-3.02]	2.57 [2.42-2.98]	0.745	0.813
LC-SFA (>C16)	40.42 [38.76-42.34]	42.37 [41.28-43.77]	0.034	0.102
MC-MUFA	1.21 [1.14-1.36]	1.15 [0.97-1.22]	0.706	0.813
LC-MUFA (>C16)	35.73 [32.60-37.62]	35.44 [34.28-36.44]	0.603	0.804
ω3 PUFA	1.77 [1.60-1.86]	1.30 [1.17-1.40]	0.002	0.010
ω6 PUFA	13.55 [12.62-15.39]	14.55 [13.61-14.82]	0.273	0.623
ω3/ω6 PUFA ratio	0.13 [0.12-0.14]	0.09 [0.08-0.10]	0.001	0.007

B. At 30 days of lactation

Fatty acid (%)	N group (n=10)	O group (n=14)	p-value	Adjusted p-value
C10:0	0.17 [0.15-0.21]	0.13 [0.07-0.18]	0.057	0.114
C12:0	0.65 [0.53-0.76]	0.33 [0.25-0.44]	0.011	0.042
C14:0	3.03 [2.69-3.50]	2.26 [1.95-2.48]	0.000	0.000
C15:0	0.39 [0.38-0.43]	0.40 [0.37-0.42]	0.941	0.991
C16:0	24.14 [23.47-24.72]	22.66 [22.37-23.01]	0.000	0.001
C18:0	14.55 [13.88-14.78]	16.22 [14.92-16.62]	0.027	0.076
C10:1	0.54 [0.44-0.64]	0.22 [0.18-0.27]	0.022	0.073
C16:1ω7	3.84 [3.71-4.11]	3.91 [3.69-4.37]	0.686	0.854
C18:1ω7	1.72 [1.58-1.91]	1.86 [1.76-1.97]	0.093	0.169
C12:1	0.17 [0.15-0.20]	0.13 [0.10-0.17]	0.000	0.001
C14:1ω5	0.25 [0.19-0.26]	0.26 [0.22-0.28]	0.725	0.854
C15:1ω9	0.34 [0.27-0.38]	0.40 [0.32-0.43]	0.824	0.915
C16:1ω9	0.51 [0.47-0.59]	0.49 [0.47-0.54]	0.594	0.792
C18:1ω9	10.71 [10.27-11.26]	11.51 [10.91-11.96]	0.034	0.079
C20:1ω9	0.19 [0.15-0.21]	0.13 [0.12-0.15]	0.000	0.002
C18:3ω3	2.81 [2.53-3.73]	3.32 [2.75-3.99]	1.000	1.000
C20:3ω3	0.07 [0.06-0.09]	0.08 [0.07-0.10]	0.321	0.493
C20:5ω3	0.67 [0.63-0.80]	0.71 [0.65-0.93]	0.176	0.294
C18:2ω6	32.17 [31.39-33.08]	32.72 [30.97-34.84]	0.510	0.729
C20:4ω6	0.57 [0.52-0.66]	0.66 [0.56-0.70]	0.036	0.079
SFA	43.36 [42.27-44.04]	41.74 [40.70-42.39]	0.003	0.019
MUFA	18.71 [17.99-19.60]	18.90 [18.49-19.17]	0.804	0.941
PUFA	36.89 [36.09-37.19]	37.64 [36.63-39.30]	0.072	0.217
MC-SFA	4.19 [3.75-4.79]	3.12 [2.76-3.52]	0.000	0.000
LC-SFA (>C16)	38.88 [37.85-39.24]	38.58 [37.70-39.22]	0.745	0.941
MC-MUFA	1.48 [1.24-1.65]	1.03 [0.88-1.11]	0.006	0.026

LC-MUFA (>C16)	17.49 [16.43-17.75]	18.04 [17.24-18.49]	0.103	0.247
ω3 PUFA	3.62 [3.22-4.37]	4.39 [3.61-4.99]	0.922	0.941
ω6 PUFA	32.76 [32.00-33.69]	33.39 [31.50-35.57]	0.495	0.831
ω3/ω6 PUFA ratio	0.11 [0.10-0.13]	0.13 [0.10-0.15]	0.941	0.941

C. At 90 days of lactation

Fatty acid (%)	N group (n=10)	O group (n=14)	p-value	Adjusted p-value
C10:0	0.15 [0.12-0.33]	0.14 [0.10-0.15]	0.101	0.144
C12:0	1.05 [0.77-1.20]	0.56 [0.46-0.66]	0.000	0.000
C14:0	3.36 [3.26-3.49]	2.36 [2.28-2.82]	0.000	0.000
C15:0	0.54 [0.52-0.58]	0.47 [0.44-0.53]	0.021	0.049
C16:0	21.82 [21.30-22.08]	22.27 [21.51-22.38]	0.088	0.144
C18:0	14.10 [13.35-15.12]	15.36 [14.96-16.17]	0.009	0.025
C10:1	0.48 [0.42-0.52]	0.26 [0.23-0.34]	0.001	0.004
C16:1ω7	4.15 [3.96-4.79]	3.88 [3.76-4.05]	0.022	0.049
C18:1ω7	1.58 [1.43-1.66]	1.85 [1.74-1.93]	0.000	0.000
C12:1	0.19 [0.17-0.22]	0.16 [0.15-0.18]	0.462	0.543
C14:1ω5	0.48 [0.46-0.54]	0.52 [0.43-0.57]	0.380	0.475
C15:1ω9	0.49 [0.41-0.54]	0.35 [0.31-0.40]	0.001	0.003
C16:1ω9	0.51 [0.46-0.59]	0.49 [0.46-0.53]	0.824	0.843
C18:1ω9	9.78 [9.30-9.89]	10.21 [9.92-10.63]	0.122	0.163
C20:1ω9	0.16 [0.14-0.23]	0.15 [0.14-0.19]	0.095	0.144
C18:3ω3	3.94 [3.37-4.63]	3.45 [3.16-3.68]	0.007	0.023
C20:3ω3	0.06 [0.05-0.09]	0.06 [0.05-0.08]	0.843	0.843
C20:5ω3	2.12 [1.96-2.71]	1.96 [1.50-2.41]	0.094	0.144
C18:2ω6	31.92 [31.23-32.93]	33.37 [32.09-34.13]	0.092	0.144
C20:4ω6	0.69 [0.58-0.76]	0.66 [0.63-0.73]	0.824	0.843
SFA	40.72 [39.95-41.31]	41.56 [41.35-41.71]	0.039	0.077
MUFA	18.32 [16.96-18.99]	17.60 [17.19-18.10]	0.667	0.800
PUFA	39.13 [37.75-39.77]	38.96 [38.59-39.74]	0.922	0.941
MC-SFA	5.11 [4.76-5.44]	3.65 [3.41-4.10]	0.000	0.000
LC-SFA (>C16)	35.55 [34.56-36.64]	37.42 [37.24-38.16]	0.000	0.000
MC-MUFA	1.63 [1.43-1.87]	1.26 [1.19-1.31]	0.000	0.001
LC-MUFA (>C16)	16.79 [15.95-17.15]	16.56 [16.18-16.92]	0.941	0.941
ω3 PUFA	5.61 [5.17-6.64]	4.88 [4.67-5.07]	0.002	0.004
ω6 PUFA	32.67 [31.85-33.68]	34.00 [32.80-34.75]	0.092	0.138
ω3/ω6 PUFA ratio	0.17 [0.15-0.21]	0.14 [0.13-0.15]	0.000	0.001
